# Specialty choice determinants among Mexican medical students: a cross-sectional study

**DOI:** 10.1186/s12909-019-1830-5

**Published:** 2019-11-14

**Authors:** Carlos Gutiérrez-Cirlos, J. Jesús Naveja, Manuel García-Minjares, Adrián Martínez-González, Melchor Sánchez-Mendiola

**Affiliations:** 1 National Institute of Medical Sciences and Nutrition “Salvador Zubirán”, Mexico City, Mexico; 2 Faculty of Medicine, National Autonomous University of Mexico (UNAM), Mexico City, Mexico; 3 Coordinador de Desarrollo Educativo e Innovación Curricular, Universidad Nacional Autónoma de México, Circuito Centro Cultural, S/N. Edificio CIPPS, 1er Piso, Ciudad Universitaria, Del. Coyoacán, Ciudad de México, CdMx, 04510 México

**Keywords:** Residency training, Career choice, Professional identity, Graduate studies, México

## Abstract

**Background:**

The choice of medical specialty is related to multiple factors, students’ values, and specialty perceptions. Research in this area is needed in low- and middle-income countries, where the alignment of specialty training with national healthcare needs has a complex local interdependency. The study aimed to identify factors that influence specialty choice among medical students.

**Methods:**

Senior students at the National Autonomous University of Mexico (UNAM) Faculty of Medicine answered a questionnaire covering demographics, personal experiences, vocational features, and other factors related to specialty choice. Chi-square tests and factor analyses were performed.

**Results:**

The questionnaire was applied to 714 fifth-year students, and 697 provided complete responses (response rate 81%). The instrument Cronbach’s alpha was 0.8. The mean age was 24 ± 1 years; 65% were women. Eighty percent of the students wanted to specialize, and 60% had participated in congresses related to the specialty of interest. Only 5% wanted to remain as general practitioners. The majority (80%) wanted to enter a core specialty: internal medicine (29%), general surgery (24%), pediatrics (11%), gynecology and obstetrics (11%) and family medicine (4%). The relevant variables for specialty choice were grouped in three dimensions: personal values that develop and change during undergraduate training, career needs to be satisfied, and perception of specialty characteristics.

**Conclusions:**

Specialty choice of medical students in a middle-income country public university is influenced by the undergraduate experience, the desire to study a subspecialty and other factors (including having skills related to the specialty and type of patients).

## Background

The professional path of health professions students involves many high-stakes decisions. These begin with the choice to study medicine, then selecting a medical school, followed by the decision to enter a specialty [1]. These major turning points have an essential impact on a physician’s personal and professional life and, ideally, they should not be taken lightly or with limited information. Several intrinsic or extrinsic factors influence the specialty choice of medical students, which are positively or negatively related to that choice. Bland and Meurer described a model about specialty choice in medicine that was recently reviewed and updated [2, 3]. In this model, five main categories were identified: type of medical school, student characteristics, student values, career needs to be satisfied, and perception of specialty characteristics [2]. The country of residence, place of residence in the country, socioeconomic level, personal income and the particular characteristics of the selection process in each country, are also determinant of how physicians choose a specialty or another occupation when they finish their medical career [4]. On the other hand, factors that negatively influence and motivate to reject some specialties have been identified (e.g., when general surgery is rejected because of harassment and negative role models) [5–8].

Despite the importance and lifelong relevance of specialty choice in medicine, there is relatively little research in this area, particularly in low- and middle-income countries [9]. This is noteworthy since emerging economies frequently have issues of misalignment among some of the following elements: the creation of new medical schools, size of admission cohorts, capabilities and limitations of clinical sites to provide specialty training, healthcare specialty needs of the national health system and geographical distributions of the graduated specialists. There is, therefore, an urgent need for current and relevant data in order to make more evidence-based decisions, otherwise medical students will continue following gut feelings, tradition, or familial preferences.

The objective of this study was to evaluate factors influencing specialty choice among senior medical students. Demographic factors, curricular exposure (e.g., teaching, research, primary care or surgical procedures), and expectations about the chosen specialty were considered. The exploration and understanding of the specialty choice process could contribute to explain why some in-demand specialties are not attractive to medical students.

## Methods

### Study design and setting

The study was a cross-sectional exploration of the factors related to medical specialty choice among medical students at the end of their medical training in a major public university in Mexico City. We applied the questionnaire to students at the end of the fifth year of undergraduate medical training at the National Autonomous University of Mexico (UNAM) Faculty of Medicine. This medical school is the largest in the country, with more than 7000 medical students and more than 10,000 specialty residents. The MD program at UNAM lasts six and a half years and is basically free [10]. The first 2 years cover basic sciences knowledge, and the following three and a half years include clerkships and clinical training, with a one-year in-house internship in core medical areas (pediatrics, surgery, emergency medicine, ob-gyn, internal medicine, and family medicine). During the last year of medical training, all students must provide one-year of social service, mostly at primary care facilities all over the country.

### Sample

A total of 956 students were registered in the fifth year of the medical school, and 864 of them underwent a summative objective structured clinical exam (OSCE) before the social service rotation. In the OSCE stations that had no actual test material, students were asked to answer the questionnaire voluntarily. The instrument was returned by 714 students; questionnaires with less than 80% completion were excluded, with a total of 697 (81%) remaining for analysis.

### Questionnaire

The questionnaire (Additional file 1) was designed to evaluate acknowledged factors influencing the choice of a medical specialty. The process of instrument development was as follows: a literature review about instruments related to the construct of interest was performed in the major databases; the identified questionnaires were evaluated and selected by a group of experts (clinicians with experience and graduate degrees in education); the group selected the appropriate items; feedback was obtained about the items’ quality and clarity from a group of scholars at the Department of Medical Education, UNAM Faculty of Medicine; upon group consensus the final instrument was integrated to provide content validity [4, 11, 12]. The final version included 35 items, divided into two sections: a) demographic features (18 items), including whether they chose or not a core specialty (general surgery “GS”, gynecology and obstetrics “OBG/GYN”, internal medicine “IM”, family medicine “FM” and pediatrics “Peds”), in which case they were asked if the specialty was predominantly surgical or medical; and b) factors related to specialty selection (17 items), this section used a Likert-like scale, from non-determinant (zero) to most determinant (three). The second section was focused on the experiences related to the chosen specialty during medical school and the expectations in terms of academic and lifestyle features. All questionnaires were voluntarily and anonymously answered in printed form with an op-scan answer sheet. The response time for the instrument was less than 15 min.

### Data analysis

Descriptive statistics were obtained. For categorical variables, chi-square independence tests were performed. For the second section of the instrument, factor analysis was done to sort factors according to how determinant they were in choosing a specialty. Principal Components Analysis (PCA) was done to identify clusters of correlated variables. Kaiser varimax normalization was used as rotation method. Statistical analysis was performed in R version 3.4.1 and IBM SPSS 20.0.

### Ethical aspects

The study was in compliance with the Declaration of Helsinki for research involving human subjects. Review and approval were provided by the Medical Education Department in the medical school as a non-invasive minimal risk study. Participants were informed of the purpose of the study and provided verbal informed consent. Data were managed anonymously in a confidential manner.

## Results

### Demographics

The questionnaire was completed by 697 students, a response rate of 81%. The mean age of the participants was 24 ± 1 years (mean ± SD). Sixty-five percent were women. The majority (97%) were single and with no children. Regarding the parents’ educational level, in 60% of the sample, at least one parent had undergraduate studies, while in 15% at least one had postgraduate studies. In our sample, 91% of the students’ parents were not physicians. At UNAM, students have access to free medical training, whether they attended public or private High Schools; in our sample, 82% studied in a public High School.

### Previous experiences influencing specialty choice

More than half (60%) of the students had participated in congresses related to their specialty of interest, but less than a quarter (22%) had ever received structured information about the residency programs available (such as vocational counseling and career orientation). Roughly a third (30%) participated in research during their training, and 20% had been involved in teaching.

### Vocational features at the end of the medical training

About 80% of the students plan to specialize, while 12% would like other postgraduate training (a master’s or a Ph.D. program). Only 5% would prefer to remain as general practitioners, 2% would like to practice teaching and 1% other activities. Almost half (45%) were interested in practicing medicine in rural communities, independently of whether they planned to specialize or not.

About 80% of the respondents want to do a “core” specialty (i.e., internal medicine, general surgery, pediatrics, gynecology and obstetrics, or family medicine), in the following order: IM (29%), GS (24%), Peds (11%), OB/GYN (11%) and FM (4%).

### Determinants for the choice of a medical specialty

We analyzed the association of the previously discussed variables and the chosen specialty with a chi-squared hypothesis test for independence. There were statistically significant associations (*p* ≤ 0.05) only for sex and the activity at the end of the career. There was a higher proportion of men who chose GS (40% vs. 25%), while there were more women in OB/GYN (16% women vs. 9% men) and Peds (16% women vs. 10% men). For IM and FM, there were no critical differences in the proportions of women and men (37% vs. 38 and 6% vs. 3%, respectively).

In general, the most important factors for specialty choice at the end of the undergraduate medical training were the kind of work expected at the end of the specialty, the type of patients, personal ability, variety of medical problems, and the possibility of studying a subspecialty. Less determinant factors were research opportunities, role models, prestige, and financial aspects (Table 1).

**Table 1:** Average importance of each item compared by chosen specialty and sex in senior medical students at UNAM Faculty of Medicine in Mexico City (*n* = 697). 0 = not determinant, 3 = most determinant. Numbers in parentheses represent the order of importance of each item

Item	GS^a^	OB/GYN	IM	FM	Peds	Women	Men
Type of patients in the specialty	2.2 (5)	2.5 (1)	2.5 (1)	2.0 (5)	2.5 (1)	2.4 (2)	2.3 (2)
Variety of medical problems in the specialty	2.1 (6)	2.1 (5)	2.5 (2)	1.9 (10)	2.2 (8)	2.2 (4)	2.2 (6)
Work to do during the specialty	2.2 (4)	2.4 (3)	2.4 (3)	2.3 (2)	2.5 (2)	2.4 (1)	2.3 (3)
Specialty social engagement	1.8 (10)	2.0 (9)	2.1 (9)	1.9 (9)	2.2 (6)	2.0 (9)	1.9 (9)
Possibility of studying a subspecialty	2.2 (2)	2.1 (7)	2.2 (7)	1.6 (13)	2.2 (7)	2.1 (7)	2.2 (5)
Opportunities to perform research	1.6 (14)	1.3 (15)	1.6 (13)	1.4 (17)	1.5 (14)	1.5 (14)	1.7 (13)
Specialty duration	1.9 (12)	1.5 (13)	1.5 (16)	2.1 (4)	1.8 (12)	1.7 (13)	1.6 (14)
Expectation of free time	1.6 (15)	1.6 (12)	1.7 (12)	1.9 (9)	1.8 (13)	1.8 (12)	1.7 (12)
Possibility of raising a family	1.8 (11)	1.8 (10)	1.7 (12)	1.9 (9)	1.9 (10)	1.8 (10)	1.8 (10)
Potential autonomy after graduation	2.0 (8)	2.1 (8)	2.1 (8)	1.9 (12)	2.0 (9)	2.0 (8)	2.1 (7)
Financial reasons	1.4 (17)	1.5 (14)	1.3 (17)	1.4 (15)	1.4 (16)	1.3 (17)	1.4 (17)
Family support during the specialty	1.8 (9)	1.8 (11)	1.8 (10)	2.0 (6)	1.9 (11)	1.8 (11)	1.8 (11)
Role models	1.5 (16)	1.3 (17)	1.6 (14)	1.5 (14)	1.5 (15)	1.4 (15)	1.6 (15)
Pleasant academic experience in the specialty	2.2 (4)	2.1 (6)	2.3 (5)	2.2 (3)	2.3 (4)	2.2 (5)	2.3 (4)
Medical internship with pleasant experiences in the specialty	2.1 (7)	2.1 (4)	2.2 (6)	1.9 (12)	2.3 (5)	2.1 (6)	2.0 (8)
Specialty prestige	1.6 (13)	1.3 (16)	1.6 (15)	1.4 (17)	1.2 (17)	1.4 (16)	1.5 (16)
To have skills related to the specialty	2.2 (1)	2.4 (2)	2.4 (4)	2.5 (1)	2.4 (3)	2.3 (3)	2.4 (1)

^a^*GS* general surgery, *OB/GYN* gynecology and obstetrics, *IM* internal medicine, *FM* family medicine, *Peds* pediatrics

Even though financial matters were generally disregarded, students that selected OB/GYN or FM rated this factor higher than other students. Having the required skills for the specialty was generally an influencing factor, and even more for students that selected GS, FM or OB/GYN. Family support seemed more relevant for FM.

Pleasant experiences during the medical internship were slightly more relevant for GS and FM. Prestige tended to be more important for GS. Autonomy was less determinant for FM. The duration of the specialty was more relevant for FM. The duration of the FM specialty in Mexico is 3 years, while for the others it is 4 years, except for Peds, which has a duration of three to 4 years, depending on the hospital and university program [13]. This could be related to the possibility of studying a subspecialty, which seemed more important for the non-FM specialties. The possibilities of doing research were less important for OB/GYN and FM. Social aspects of the specialty had a higher priority for Peds and FM. The wide variety of medical problems faced in IM practice was determinant for choosing this specialty. Interest in the type of patient was more important for IM, Peds, and OB/GYN than for GS and FM, which could be explained by the fact that these have a large variety of patients. The characteristics of the job that is performed in the chosen specialty had a high priority for all the specialties but somewhat lower for GS. The availability of free time was less important for GS and more important for FM. Raising a family was a higher priority for FM. Role models seemed not to vary their importance in the different selected specialties.

We also identified factors that were more determinant, depending on sex. For women it was more important to consider the variety of medical problems in the specialty, the kind of work, the duration of the specialty and experiences during the clinical internship, while for men the possibility of doing research, pleasant academic experiences, to have skills related to the specialty, to have autonomy and the possibility of studying a subspecialty were more relevant. Raising a family was equally important for men as for women. Other factors similarly relevant for men and women were type of patient, social engagement, free time expectations, financial reasons, family support, role models, and prestige.

### Factor analysis

It is to be expected that some determinants of specialty choice are positively correlated, for example, free time expectations and the opportunity to raise a family. The factor analysis showed that variables could be roughly clustered in three groups: factor 1 (F1) comprises the variables “interest in the specialty patient type”, “variety of medical problems in the specialty”, “work to do during the specialty”, “specialty social engagement”, “possibility of studying a subspecialty” and “opportunities to perform research”; factor 2 (F2) the variables “specialty duration”, “expectation of free time”, “possibility of raising a family”, “potential autonomy after graduation”, “financial reasons” and “family support during the specialty” and factor 3 (F3) includes the variables “role models”, “pleasant academic experience in the specialty”, “medical internship with pleasant experiences in the specialty”, “specialty prestige” and “to have skills related to the specialty” (Table 2).

**Table 2:** Factor analysis^a^ of determinants of specialty preference in senior medical students at UNAM Faculty of Medicine in Mexico City (*n* = 697). The last column shows the mean importance of the item (0 = not determinant; 3 = most determinant)

Item	Personal values during undergraduate training (F1)	Career needs to be satisfied (F2)	Perception of the specialty characteristics (F3)	Mean importance
Interest in the specialty patient type	0.77	–	–	2.3
Variety of medical problems in the specialty	0.74	–	–	2.2
Work to do during the specialty	0.69	–	–	2.4
Specialty social engagement	0.60	–	–	2.0
Possibility of studying a subspecialty	0.50	–	–	2.1
Opportunities to perform research	0.40	–	–	1.6
Specialty duration	–	0.77	–	1.7
The expectation of free time	–	0.76	–	1.8
Possibility of raising a family	–	0.72	–	1.8
Potential autonomy after graduation	–	0.58	–	2.0
Financial reasons	–	0.44	–	1.4
Family support during the specialty	–	0.41	–	1.8
Role models	–	–	0.68	1.5
Pleasant academic experience in the specialty	–	–	0.63	2.2
Medical internship with pleasant experiences in the specialty	–	–	0.62	2.1
Specialty prestige	–	–	0.57	1.4
To have skills related to the specialty	–	–	0.42	2.2
Alpha	0.73	0.71	0.65	–
Variance explained	24.0%	12.4%	8.3%	–
Cumulative variance explained	24.0%	36.4%	47.7%	–

^a^Extraction method: principal components. Rotation method: Kaiser varimax normalization. Factor loading considered ≥0.4

**Fig. 1:**
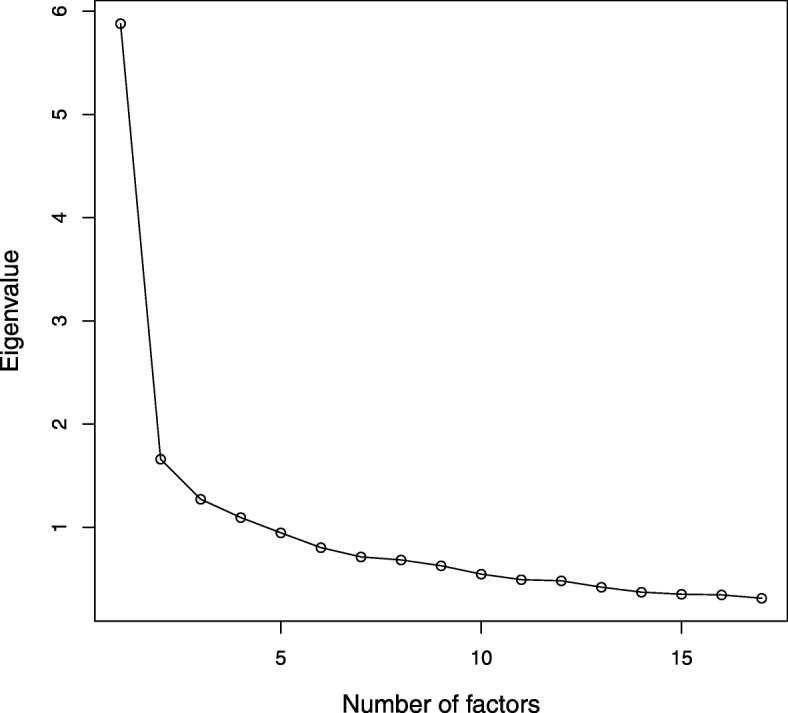
Scree plot showing the variance contribution of 17 factors for specialty choice in senior medical students, UNAM Faculty of Medicine in Mexico City (n = 697). The first three (F1 to F3) explain 47.7% of the total variance

The figure shows a scree plot with the variance contribution of the 17 factors identified in the analysis. As mentioned earlier, only the first three factors (F1-F3) were further considered in the analysis, as they explain 47.7% of the total variance (Fig. 1).

## Discussion

Most physicians in Mexico choose specialties such as GS, OB/GYN, IM, and Peds, even though there is a paradoxically decreasing job offer for these graduates [14]. Nonetheless, in-demand medical specialties for the epidemiologic landscape of the country like geriatrics and radio-oncology have limited appeal. In a recent study of first-year core specialties’ Mexican residents, a comprehensive questionnaire was applied to identify factors that have an influence on the specialty choice [11]. The findings showed that choices were made during the last years of undergraduate training and that the type of patient was determinant for choosing Peds, while a well-defined academic program was determinant for internal medicine. The dimensions found in our factor analysis resemble Bland-Meurer classification: personal values that develop and change during the undergraduate training (F1), career needs to be satisfied (F2), and perception of specialty characteristics (F3) [2, 3]. This confirms the finding that students choose a specialty based on a “package” or “cluster” of characteristics, rather than single features or interest in the specialty [15].

Given the features of the specialty programs and healthcare systems in low- and middle-income countries, free time availability is limited in almost all specialties, and may not be a determinant for residency selection [9]. Nonetheless, in other countries, free time and lifestyle have been found as an important specialty selection determinant [16–18]. A controllable lifestyle, financial factors, and free time were found to be important factors in medical students and doctors in a Brazilian study [4]. Other fundamental aspects are personality traits [11, 19–22], the possibility of further studying a subspecialty program [11], and the specialty prestige [17, 23, 24]. It has been previously reported that if the parents are physicians, this could also influence specialty choice [9].

Similar to the findings of Cleland, favorable working conditions were among the most prominent features for selecting a residency position [12]. Furthermore, women deemed this feature even more important than men, also in agreement with our results. As previously reported, the coverage of positions for primary care specialties has diminished as a result of a reduction in the number of students that select these specialties. Exposing students to academic experiences related to primary care seems not to modify choice rates [25].

Most medical students reach a final specialty decision after they undergo clinical training in medical school, typically during the last clinical immersion year [3, 9]. We found that 38% reached a final specialty decision during the medical internship (fifth year in our program); followed by 27% who decided during undergraduate studies but before the internship; 20% who hadn’t decided at the end of the medical internship; 10% decided before admission to medical school, and 5% after finishing the medical internship. As a consistent finding, surgeons decide their specialty choice earlier, even before entering medical school. In our study, 13% of the students who chose general surgery reached their decision before undergraduate studies, in contrast to 10, 6, 4 and 12% in OB/GYN, IM, Peds, and FM, respectively.

The complex situation of admission in a specialty residency in Mexico has been commented elsewhere [14, 26]. In 2016 only 20% of the applicants were selected from a pool of almost 40,000, and 51% were enrolled in a course focused on achieving higher scores in the national residency exam. We also found a trend of more women in medical than surgical specialties as compared to men; this is maintained even when they chose not to follow a non-core specialty. These results agree with many other previous reports that show a trend towards an increasing feminization of medical practice and some specialties [27, 28]. Men tend to choose surgical specialties more often than women, as has been documented [6–9, 11, 22, 29].

Although it is evident that most students that choose a core medical specialty think that their primary activity will be related to that specialty, some considered other activities such as other postgraduate courses, a teaching career or remaining as general practitioners. This was notorious in students who chose FM, where 45% consider these activities, in contrast to those who chose other specialties (only 20% of the sample considered these other activities).

In a country with an epidemiological profile that has changed towards chronic degenerative diseases, the sociodemographic factors and the organization of the health systems should influence the vocational choice during medical studies. Despite the increased demand of primary care physicians, interest in other specialties that have a direct entrance (such as geriatrics, psychiatry or radiology) is still not enough to cover the need for such specialists. Early exposure with positive experiences related to highly demanded specialties according to each country’s health system is important to modify the specialty choice and meet the training needs in other specialties.

One of the limitations of the study is that it was done in only one medical school, although the large sample and the characteristics of the Faculty of Medicine (public school, largest in the country, students from all the Mexican states, population similar to other public medical schools) provide strength to our findings. This is the first reported use of the study’s questionnaire, and even though we have information from several sources of validity evidence (mostly regarding internal structure and content), we are aware that there is a need to continue accumulating evidence from other sources and to apply the instrument in different settings to other populations.

The study was performed at the end of the medical school career where most students have clarity about their career choices, although the low acceptance rate in the residency selection process in Mexico can compel many of these students to change their final vocational choice or opt for a “less than ideal” specialty that is less competitive. The variety of issues that determine the quality of professional trajectory decisions in medicine generate a field ripe for research studies. One of the most significant challenges of twenty-first century global healthcare is the asymmetry of the societal needs and the educational trajectories of its healthcare professionals, this situation needs to be addressed with quantitative and qualitative research to better understand the nuances of the decision-making process, at the individual and institutional levels, and offer credible and practical solutions. Stakeholders in medical education and healthcare communities should inform students about specialties’ pros and cons in a balanced manner and attempt to influence modifiable factors in specialty choice.

## Conclusion

In Mexico, similar to other countries, there is a tendency towards specialization in medicine. Most students prefer a medical or surgical specialty program, mainly in the core specialties. A majority of students decide on specialty choice during the last years of undergraduate training. Pleasant academic experiences and the characteristics of the medical internship are determinants in specialty choice. Overall, personal values (e.g., personal preferences and positive attitude towards patients) and the perceived characteristics of the specialty (e.g., related work, pleasant academic experience) are more determinant than career needs to satisfy (e.g., financial reasons, prestige).

## Supplementary information

**Additional file 1.** Factors related to specialty selection questionnaire.

## Data Availability

The datasets used in the study are available from the corresponding author on request.

## Abbreviations

F: Factor (from the factor analysis)
FM: Family medicine
GS: General surgery
IM: Internal medicine
OB/GYN: Gynecology and obstetrics
OSCE: Objective structured clinical exam
PCA: Principal components analysis
Peds: Pediatrics
SD: Standard deviation
UNAM: National Autonomous University of Mexico
