# Supplementary material for: Specialty choice determinants among Mexican medical students: a cross-sectional study
Source: BMC Med Educ. 2019 Nov 14;19:420. doi: 10.1186/s12909-019-1830-5 (PMC6854711; doi:10.1186/s12909-019-1830-5)
Supplement: Supplementary file 1 — Additional file 1. Factors related to specialty selection questionnaire. [file 12909_2019_1830_MOESM1_ESM.docx]

## Survey about factors related to specialty residency selection

## National Autonomous University of Mexico (UNAM) Faculty of Medicine

**Mexico City, Mexico**

The **objective** of this survey is to explore some factors possibly related with the choice of postgraduate studies (specialty, Masters or PhD programme) after finishing undergraduate medical studies. The survey is anonymous and filling it will require less than 10 minutes. **Thank you for your participation.**

Answer each of the following questions by filling in the circles in the answers sheet accordingly:

**1.- Sex**

1. Female
2. Male

**2.- Marital status**

1. Single
2. Married
3. Separated/divorced with children
4. Separated/divorced with no children
5. Widow

**3.- Educational level of mother:**

1. Elementary or less
2. Jr. High School
3. High School
4. Undergraduate
5. Postgraduate (specialty, Masters or PhD)

**4.- Educational level of father:**

1. Elementary or less
2. Jr. High School
3. High School
4. Undergraduate
5. Postgraduate (specialty, Masters or PhD)

**5.- Is your parent a physician?**

1. No
2. Yes, he/she is a general practitioner
3. Yes, he/she studied a **different** specialty as the one I'm interested in.
4. Yes, he/she studied at **the same** specialty as the one I'm interested in.

**6.- To what kind of High School did you attend?**

1. Public
2. Private

**If you studied in a private High School, skip to question 8.**

**7.- If you attended a public High School specify:**

1. National High School
2. Sciences and Humanities College
3. Other

**8.- What version of UNAM Faculty of Medicine curriculum did you register for?**

1. 2010
2. 2010 - PECEM
3. Other (Unique, Plan 85)

**9.- Have you participated in study groups or assisted to congresses related to the specialty you are interested?**

1. Yes
2. No

**10.- Have you received structured information (counseling, vocational counseling, conferences, articles) related to the medical residencies programmes in Mexico?**

1. Yes
2. No

**11.- Have you participated in research (biomedical, clinical, public health, education) during your medical studies?**

1. Yes
2. No

**12.- Have you participated in teaching activities as an instructor or teacher assistant recognized by the University?**

1. Yes
2. No

**13.- When did you decide to choose a specialty, in case you have already reached such decision? *(fill option A if you have not reached a decision yet).***

1. I haven't chosen a specialty yet
2. Prior to entering Medical School
3. During the first four and a half years of Medical School, but prior to the clinical internship
4. During the clinical internship
5. After the clinical internship

**14.- After finishing your undergraduate training as a physician, what will be your main activity?**

1. Working as a general practitioner
2. Teaching
3. Studying a medical specialty
4. Other postgraduate courses (Masters or PhD)
5. Other

**15.- Would you work in a rural area or small community, once you have obtained your medical license?**

1. Yes
2. No

**16.- If you chose a core specialty, what would be your first option?** ***(Don't answer this question if you do not consider pursuing a core specialty)***

1. General surgery
2. Gynecology
3. Internal medicine
4. Family medicine
5. Pediatrics

**17.- If you have chosen a non-core specialty, what of the following possibilities have you considered? *(Don't answer this question if you do not consider pursuing a core specialty)***

1. Clinical (dermatology, psychiatry, anesthesiology, imageology, cardiology, etc)
2. Surgery (ORL, ophthalmology, orthopedics, plastic surgery, etc)

**18.- Will you attend a training course for the National Exam for Candidates to Medical Residencies (ENARM)?**

1. Yes
2. No

**Rate each of the following factors according to how determinant it is for the choice of a medical specialty, from A (-) to D (+++) (*where A would be non-determinant and D most determinant)*:**

| **Factor** | **A**  **-** | **B**  **+** | **C**  **++** | **D**  **+++** |
| --- | --- | --- | --- | --- |
| 19.- Economical reasons |  |  |  |  |
| 20.- Knowing to be skilled or dexterous for the chosen specialty |  |  |  |  |
| 21.- Adequate academic experience in such specialty |  |  |  |  |
| 22.- Good experiences in the clinical internship in such specialty |  |  |  |  |
| 23.- Role models |  |  |  |  |
| 24.- Prestige of the specialty |  |  |  |  |
| 25.- Potential autonomy (independency after the studies) |  |  |  |  |
| 26.- Length of the specialty |  |  |  |  |
| 27.- Family support during the specialty |  |  |  |  |
| 28.- Opportunities to carry out research |  |  |  |  |
| 29.- Social commitment of the specialty |  |  |  |  |
| 30.- Variety of medical problems related to the specialty |  |  |  |  |
| 31.- Interest in the type of patient associated with the specialty |  |  |  |  |
| 32.- The duties associated with the specialty |  |  |  |  |
| 33.- The possibility of having free time |  |  |  |  |
| 34.- The possibility of raising a family |  |  |  |  |
| 35.- The possibility of studying a subspecialty |  |  |  |  |

**Thanks for your participation.**
